# Draft Genome Sequences of Seven 4-Formylaminooxyvinylglycine Producers Belonging to the *Pseudomonas fluorescens* Species Complex

**DOI:** 10.1128/genomeA.00277-17

**Published:** 2017-05-04

**Authors:** Rachel A. Okrent, Viola A. Manning, Kristin M. Trippe

**Affiliations:** a USDA-ARS National Forage Seed Production Research Center, Corvallis, Oregon, USA; b Department of Crop and Soil Science, Oregon State University, Corvallis, Oregon, USA

## Abstract

Vinylglycines are nonproteinogenic amino acids that inhibit amino acid metabolism and ethylene production. Here, we report the draft genome sequences of seven isolates of *Pseudomonas* that produce 4-formylaminooxyvinylglycine, a compound known to inhibit the germination of grasses and the growth of specific plant-pathogenic bacteria.

## GENOME ANNOUNCEMENT

The *Pseudomonas fluorescens* species complex is a particularly diverse group of bacteria that play important ecological functions in soil, plant, and animal systems. These ecological and agricultural functions, including plant growth promotion, induction of systemic resistance, and biological control, are often mediated by secondary metabolites (1–4). One compound that contributes to the utility of *P. fluorescens* is the nonproteinogenic amino acid 4-formylaminooxyvinylglycine (FVG) (5, 6). FVG has both herbicidal and antimicrobial properties (7, 8). The activity, production, and regulation of FVG have been studied in the sequenced strain *P. fluorescens* WH6 (9–12). However, little is known about the production of FVG in other strains of *P. fluorescens*.

In order to add to our understanding of the evolution and diversity of FVG biosynthesis, the genomes of seven additional FVG producers have been sequenced. These strains were isolated from environmental sources in the central Willamette Valley of Oregon, including the rhizosphere of grasses (*Poa* spp. and *Lolium perenne* L.) (6, 7, 13) and the surface of a basidiomycete.

Genomic DNA was extracted using PowerLyzer UltraClean Microbial DNA isolation kit (Mo Bio, Inc.). Sequencing libraries were prepared using the Nextera XT DNA library preparation kit (Illumina). High-throughput sequencing MiSeq runs were performed on a standard flow cell version 3, with 300-bp paired-end reads, at the Center for Genome Research and Biocomputing at Oregon State University. The reads were trimmed and assembled into contigs using CLC Genomics Workbench versions 8.0.3 and 8.5.1 (Qiagen) and manually inspected for quality. Contigs less than 500 nucleotides (nt), those less or more than ~3× average coverage, or those composed primarily of reads that could not be unambiguously placed were discarded. The remaining contigs were aligned to a reference sequence using the Move Contigs function (14) in Mauve version 2015-02-25 (15). Scaffolds were confirmed and filled in with sequence when possible, and additional contigs were discarded if they did not align to the reference and did not contain coding regions. The reordered multi-FASTA files for the draft genomes were annotated using the NCBI Prokaryotic Genome Annotation Pipeline. Genes related to FVG production were manually reannotated. The characteristics and accession numbers of the draft genomes are summarized in Table 1. Pairwise average nucleotide identity (ANI) analysis was performed using the Microbial Species Identifier tool from the Joint Genome Institute (16).

**TABLE 1:** Genome statistics

Strain	Accession no.	Genome size (Mb)	No. of contigs	*N*_50_ (kb)	Mean coverage (×)	No. of CDSs^a^	G+C content (%)
AH4	MSDE00000000	6.40	41	346	255	5,743	60.5
A3422A	MSDK00000000	6.14	44	292	186	5,539	60.5
TDH40	MSDG00000000	6.14	56	233	104	5,545	60.5
G2Y	MSDJ00000000	6.14	29	345	248	5,534	60.5
P5A	MSDF00000000	6.83	66	241	173	6,091	60.2
E24	MSDI00000000	6.41	42	262	141	5,782	60.3
A342	MSDH00000000	6.12	46	274	217	5,339	59.8

a CDSs, coding sequences.

Based on a comparison of pairwise ANI values, the sequenced isolates are predicted to belong to four different cliques within the *Pseudomonas fluorescens* species complex. Analysis of the gene contents of the strains revealed that all seven strains contain two chromosomal regions linked to the production of FVG (10). One is the *prtI*-*prtR* sigma factor/anti-sigma pair, which regulates FVG production in the strain *P. fluorescens* WH6 (11). The second is the 13-kb *gvg* cluster, containing multiple genes required for the production and transport of FVG (12).

### Accession number(s).

The whole-genome shotgun projects have been deposited at DDBJ/EMBL/GenBank under the accession numbers listed in Table 1. The versions described in this paper are the first versions of each project.
